# Natural infection as a blueprint for rational HIV vaccine design

**DOI:** 10.1080/21645515.2016.1232785

**Published:** 2016-09-20

**Authors:** Marlies M. van Haaren, Tom L. G. M. van den Kerkhof, Marit J. van Gils

**Affiliations:** Laboratory of Experimental Virology, Department of Medical Microbiology, Center for Infection and Immunity Amsterdam (CINIMA), Academic Medical Center, University of Amsterdam, Amsterdam, The Netherlands

**Keywords:** broadly neutralizing antibodies, co-evolution, envelope glycoprotein, HIV-1, vaccine

## Abstract

So far, the development of a human immunodeficiency virus (HIV) vaccine has been unsuccessful. However, recent progress in the field of broadly neutralizing antibodies (bNAbs) has reinvigorated the search for an HIV vaccine. bNAbs develop in a minority of HIV infected individuals and passive transfer of these bNAbs to non-human primates provides protection from HIV infection. Studies in a number of HIV infected individuals on bNAb maturation alongside viral evolution and escape have shed light on the features important for bNAb elicitation. Here we review the observations from these studies, and how they influence the rational design of HIV vaccines.

## Introduction

The development of an effective human immunodeficiency virus (HIV) vaccine would represent a breakthrough in the battle against the ongoing HIV pandemic. However, after decades of research, an effective HIV vaccine remains elusive. Most effective antiviral vaccines currently used rely on the elicitation of neutralizing antibodies (NAbs) in the immunized individual.^1^ These NAbs can prevent virus infection by direct neutralization of the virus upon entry. Although there have been 6 large scale HIV vaccine efficacy trials to date, none of them proved to be fully effective in preventing HIV transmission.^2-9^ Nonetheless, modest partial protection was observed in the RV144 clinical trial, where non-NAbs were shown to be the correlate of protection, however the exact mechanism is still under investigation.^10-12^ To enhance future vaccine efficacy improved vaccine immunogen design strategies should be explored, for instance those utilizing knowledge obtained from natural HIV infection.

One of the main features of HIV is its high sequence variability, which causes a great diversity in the circulating HIV strains.^13,14^ As a result, the induction of strain-specific NAbs will not be adequate to prevent infection by all circulating HIV species and it is therefore highly desirable to elicit broadly neutralizing antibodies (bNAbs) by vaccination. These bNAbs are able to neutralize a broad range of heterologous HIV and several passive transfer studies have demonstrated that full protection against HIV acquisition can be achieved by bNAbs in rhesus macaques.^15-17^ Therefore, bNAbs form important templates for HIV immunogen design. However, the question remains how to elicit these bNAbs in HIV naive, healthy individuals through vaccination.

### Antibody responses during HIV infection

HIV infection is usually established by transmission of a single virus particle, the transmitted/founder (T/F) virus.^18^ Several months post-seroconversion, NAbs will be produced, which can only neutralize autologous virus, making them strains-specific.^19-21^ Nevertheless NAbs exert a selective pressure on the Env proteins of the circulating viruses, resulting in the appearance of HIV escape mutants. These escape viruses trigger new rounds of NAb affinity maturation, which can eventually lead to the development of heterologous NAbs that are capable of neutralizing a broader range of HIV viruses in approximately 20–30% of naturally HIV-1 infected individuals.^19,20,22-27^ Usually these bNAbs arise after 2–3 y of persistent HIV infection and target more conserved areas on the HIV Env protein such as the CD4 binding site (CD4bs), V1V2 apex, gp120-gp41 interface, gp120 glycan patch, and the membrane proximal external region (MPER) of gp41.^19,25,28-36^

In recent years, technologies and methods for isolating and characterizing Abs from infected individuals have become more efficient. This has resulted in a dramatic expansion of the number of bNAbs and their target epitopes that have become available for the HIV vaccine field.^25,33,34,37-40^ It is becoming apparent that almost the entire HIV Env surface can serve as a target for various bNAbs.^41^ Nevertheless some areas of the Env protein are targeted more often and therefore are designated as (super)sites of vulnerability. bNAbs possess a number of often-shared characteristics. For example, bNAbs are usually highly mutated. The mutation frequency in the variable region of the heavy chain (V_h_) is 20–50%, which is 2–5 times more than typical Abs against other pathogens.^37,42^ This suggests that multiple rounds of affinity maturation are necessary to acquire sufficient breadth. Second, bNAbs frequently have unusually long heavy chain complementary-determining region 3 regions (HCDR3s) of up to 38 amino acids.^43,44^ Possessing longer HCDR3s is thought to provide bNAbs with the ability to penetrate the glycan shield that surrounds the conserved HIV Env protein domains.^45,46^ Lastly, bNAbs are often auto- or polyreactive.^47-50^ Auto- or polyreactivity could be advantageous to bNAbs since it might assist in increased virion binding through enhancing avidity.^51^ However, autoreactivity would not be a desired property of bNAbs induced by vaccination, since this reactivity against self-antigens could potentially result in adverse effects. In general, all these characteristics are associated with negative selection during B cell development. Yet, in HIV infected individuals, Abs with either of these properties are not subjected to negative selection in early development or after maturation of the bNAbs. This is probably due to an impaired immune system in these individuals.^47,52,53^ Therefore, it will be challenging to induce a bNAb response that displays these features in an uninfected individual with a fully functional immune system. Knowledge about the development of bNAbs in natural infection can guide rational vaccine design.^54^

### Co-evolution of virus and antibody

In general there are 2 different ways in which bNAbs can develop during natural infection. Both mechanisms depend on continuous virus evolution alongside Ab maturation. Either Abs evolve from the autologous neutralizing response, acquiring more breadth over time,^33,39^ or they are established as an independent Ab lineage after escape from an early autologous neutralizing response.^25,34,38^ An example of the former is the bNAb VRC26, directed to an N160 glycan dependent epitope at the V1/V2 apex. The germline of VRC26 was engaged by a superinfecting (SI) virus possessing the more commonly occurring N160 glycan, in comparison to the T/F virus that lacked this specific glycan.^39^ Viral escape from early VRC26 Abs facilitated affinity maturation mainly in the CDRH3 of this lineage, increasing binding and neutralization properties. Recombination between the SI and T/F virus resulted in high diversity, especially in the V1/V2 apex, of circulating viruses further increasing Ab affinity maturation and thereby increasing breadth.

In the other process, selective pressure exerted by the autologous NAb response will result in the appearance of a new epitope capable of engaging the germline of an independent bNAb lineage.^34^ Emergence of distinctive Ab lineages allows for the development of different bNAb waves during HIV infection. Combined, these bNAb waves might contribute to increased neutralization breadth and potency.^25,40,55^ In one individual described by Wibmer et al.,^25^ 3 distinctive bNAb waves, targeting the V1V2 apex, CD4bs and a still undefined epitope, contributed to neutralization breadth. Proof that bNAbs complement each other in generating increased serum breadth came from a study by Bonsignori *et al.*^55^ They demonstrated that serum neutralization could be recapitulated by combining 2 clones of distinct bNAb lineages, CH01-04 and CH30-34, targeting different epitopes. Indicating that combined, distinct bNAb lineages can complement each other to eventually create broad neutralization.^25,55^ Similar observations were made in the individual that elicited the CH103 bNAb.^33^ This individual also developed a second bNAb, CH235.^40,56^ In contrast to the development of different Ab waves with multiple specificities, CH103 and CH235 target the same area on the Env protein (the CD4 binding site), permitting CH235 specific viral escape to influence the epitope for CH103. Whether interaction with a second (or third) NAb lineage, with similar or distinct epitopes, is crucial for development of fully mature bNAbs remains to be seen. If so, this might complicate vaccine design considerably.

Interestingly, these studies also reveal that clonal family members of bNAbs often show limited neutralization capacity even though they underwent similar levels of affinity maturation.^27,44,57^ This underlines the importance to guide the Ab affinity maturation at different steps of the selection process. Very recently the developmental pathway was mapped for a bNAb response directed to the N332-glycan on the Env protein.^58^ The Ab lineage diverged very early in development in response to Env escape mutants, overall the extend of SMH was limited and the N332-directed bNAbs contained few characteristics usually associated with negative selection during B-cell development. Suggesting a pathway of bNAb development that could more easily be achieved through vaccination.

### Vaccine strategies

On HIV, the only target for NAbs is the Env protein, since this is the only protein readily accessible on the outside of the virus. Env is a trimeric protein comprised of proteolytically processed gp120 and gp41 subunits linked through a non-covalent interaction.^59-61^ The Env protein is covered in a thick layer of glycans which conceal many of its epitopes.^61^ During infection, Env can be present in different conformations. In the pre-fusion conformation, the Env protein has a compact or closed formation. This conformation is often referred to as the native form of Env and exposes the epitopes for bNAbs best. Binding to the CD4-receptor and subsequent conformational changes to facilitate membrane fusion, result in a more open conformation. In this state, epitopes for non-NAbs will be more easily accessible. Because these epitopes are generally immunodominant, an open Env conformation might divert the Ab elicitation away from the development of bNAbs. Therefore it is widely accepted that immunizations should contain Env proteins presented in the native-like conformation.

In clinical HIV vaccine trials to date monomeric, monovalent gp120 or gp140 soluble proteins have been used.^3,62-64^ Although soluble gp120 elicits a weak NAb response against neutralization sensitive (Tier 1) viruses, it does not induce the desired bNAbs.^3,62-64^ One possible reason is that many epitopes for non-NAbs are exposed that are usually obscured in a trimeric conformation and that might interfere with the induction of bNAbs, while conversely many bNAb epitopes, in particular those that depend on quaternary structure, are not present on gp120. Because autologous neutralization might be an important starting point for developing bNAbs as indicated by natural infection, first efforts were directed at eliciting such a response in immunized animals using improved immunogens. First attempts to increase NAb responses included the development of soluble gp140 Env proteins, truncated at gp41 to remove the transmembrane domain.^65,66^ In the early generations of gp140 proteins cleavage between the gp120 and gp41 subunits was prevented, usually by mutation of the canonical furin cleavage site.^67^ Furthermore, in many cases a trimerization motif was added to increase the trimerization propensity.^68,69^ However, these uncleaved gp140 proteins assume a heterogeneous aberrant conformation and therefore do not resemble the native Env protein. Immunogenicity experiments with such gp140 proteins were disappointing, yielding only non-NAbs or NAbs against Tier 1 viruses.^64,70^ Not even the autologous viruses could be neutralized. Therefore, attention has shifted to presenting the Env protein in a native-like conformation. Recently stable and correctly processed native-like HIV Env trimers have been generated, termed SOSIP proteins.^71,72^ These Env trimers contain stabilizing mutations, keeping them in their closed, native conformation. In addition to SOSIP trimers, native flexibly linked trimers (NFL-trimers) form another line of research into native-like trimers.^73^ In NFL constructs, the furin cleavage site is replaced by a flexible GS-linker, rendering the protein cleavage-independent while maintaining sufficient flexibility for the proteins to ensemble into native-like trimers. Other, more epitope-focused approaches using gp120 subunits, gp120 outer domains, or scaffolds are also being tested.^74-76^ A recent study by Sanders *et al.*^77^ showed that autologous neutralization could be induced by immunization with stable and native like BG505 SOSIP (subtype A) or B41 SOSIP (subtype B) proteins.^77^ Autologous NAb responses were seen in both rabbits and macaques. However in spite of the high autologous NAb titers, heterologous neutralization was limited to Tier 1 viruses.^77^

Because immune activation is relatively weak using single Env trimers, nanoparticles are being explored.^78^ Clustering of Env proteins on the surface of a suitable nanoparticle, such as liposomes, ferritin or lumazine, might enhance immunogenicity through increased B-cell cross-linking.^78-80^ Although important Env epitopes might be obscured by increased density. Immunizations with ferritin displayed SOSIP Envs increased the NAb titers in mice, as well as rabbits compared to soluble SOSIP Env trimers, however the responses were not broadened.^79^ Although promising, SOSIP-, NFL-trimers, and nanoparticle display, therefore need to be developed further in order for them to elicit bNAb responses.

Activating Ab germline precursors that have the ability to evolve into bNAbs might be necessary to ensure development of broad neutralization activity.^81^ However, the inferred germline versions of bNAbs do not usually bind with high affinity to native Env proteins or immunogens.^13^ Thus, in order to activate desirable germline precursors, Env immunogens should be designed that are able to engage such germline precursors. Jardine *et al*.^82^ designed an engineered gp120 outer domain (eOD) protein which was able to bind CD4bs directed bNAbs, as well as their inferred precursors, providing these immunogens with the ability to potentially engage germline bNAb lineages in humans, which was confirmed by the use of germline bNAb knock-in mice.^78,82^ Although an Ab response was observed after immunogenicity experiments in these knock-in mice, there was no neutralizing activity. This confirms that additional affinity maturation of the Ab is necessary to gain neutralization activity.

**Figure 1.**
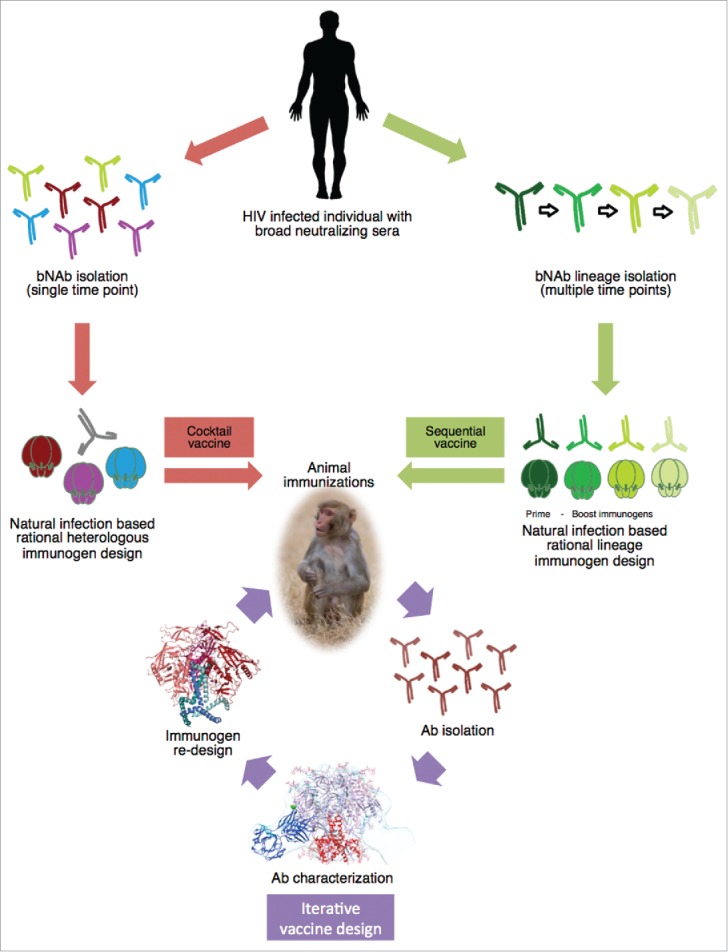
Schematic of rational HIV immunogen design based on natural infection. Env glycoprotein immunogens are rationally designed based on Abs isolated from an HIV infected individual that displays broad neutralization. Vaccine strategies entail cocktail vaccination to elicit bNAbs against a conserved site among different Env proteins. Or sequential vaccination with Env immunogens designed based on a developing bNAb lineage. Subsequent animal immunizations with the designed Env proteins and iterative vaccine design will guide optimization of the immunogen through isolation and characterization of NAbs from immunized animals, providing detailed knowledge on germline usage and target epitope. Which will in turn improve the Env protein immunogen design, improving it to elicit bNAbs.

Although it would be optimal if a single vaccination could induce an adequate protection against HIV infection, it is more likely that several booster vaccines are necessary to ensure full protection. From natural infection we know that Env diversity is positively correlated with the development of bNAbs later on in infection.^38,39,58,83^ In addition, increased viral diversity is observed in HIV infected individuals directly preceding development of breadth. Based on this knowledge vaccination with a cocktail of different HIV variants could potentially be a way to induce bNAbs (Fig. 1).^84^ Supporting the concept of a cocktail vaccine are co-evolution studies showing the importance of cooperation between different bNAb lineages with similar specificities.^40^ In addition, the notion that multiple bNAb lineages with distinct specificities contribute to extended neutralization breadth in several HIV infected individuals, underlines the need for a diverse virus population able to engage several different bNAb lineages in a vaccination scheme.^25,55^ Depending on the desired developmental pathway, the composition of the vaccine can vary from including several HIV subtypes, or immunogens specifically designed to expose a particular epitope, to only comprising virus variations from within the same HIV infected individual. In contrast, sequential immunizations with evolving Env proteins, of single or multiple subtypes, has been proposed as an alternative vaccine strategy (Fig. 1).^85^ However, it should be noted that each bNAb lineage undergoes a tailored maturation pathway and the question remains if the same germline Ab can be activated in different individuals and if so, whether this would consequently give rise to the same Ab response.^86^

Since B-cell interaction with T follicular helper cells (Tfh) is of great importance for the survival and maturation process of the B cell in the germinal center, the effect of a potential vaccine on these specific CD4+ T cells should be taken into account. Tfh cells assist follicular dendritic cells in selecting only high affinity B-cells for differentiation into plasma cells or long lived memory cells, and are important for induction of SHM.^87^ During HIV infection an expansion of the amount of Tfh cells is observed, possibly due to persistent antigenic stimulation.^88^ Resulting in increased stimulation of B cells in the germinal center, including B cells that would usually have been selected against, thus generating a higher Ab response. A positive correlation between HIV-specific Tfh cells and presence of HIV-specific NAbs has been observed.^89^ However, there seems to be a fine balance in activating Tfh cells as another study suggests that continuous Tfh cell stimulation induces the upregulation of inhibitory molecules on B cells, affecting the functionality of the Tfh cell so that no adequate B cell help can be provided.^90^ Even though Tfh cells are increasingly studied in relation to HIV infection, the current knowledge is still very limited.^91-93^ And although the targeting of Tfh cells by vaccination is definitely something to consider, more research on this subject is required.

## Conclusion

The relatively short time span in which significant steps have been made toward an HIV vaccine signifies the rapid progression of the HIV vaccine field. The studies describing the co-evolution of bNAbs and virus reviewed here show that there is not just one way of eliciting bNAbs and that it is very much a complex interplay of the humoral response and viral escape. These studies also highlight the importance of viral diversity and exposure of conserved epitopes on the Env proteins, as appearance of these features often directly precede bNAb development. It also seems likely that cooperation between several bNAb lineages is important since it generally enhances the neutralization breadth in HIV infected individuals. Whether single or multiple bNAb specificities are preferable remains unclear, as both can result in proper cross-neutralizing activity. In addition, important steps have been made with the development of stable, native-like trimers and the engagement of germline bNAbs by specifically designed Env trimers. Putting the knowledge from both natural infection and vaccine immunogen design together will further the development of improved Env immunogens and vaccine strategies toward a fully protective HIV vaccine.
